# Supplementary material for: Neurotrophins induce fission of mitochondria along embryonic sensory axons
Source: eLife. 2019 Dec 2;8:e49494. doi: 10.7554/eLife.49494 (PMC6887118; doi:10.7554/eLife.49494)
Supplement: Transparent reporting form [file elife-49494-transrepform.pdf]

1st Floor
24 Hills Road
Cambridge CB2 1JP, UK

P 01223 855340
W elifesciences.org
T @elife

eLife's transparent reporting form

We encourage authors to provide detailed information *within their submission* to facilitate the interpretation and replication of experiments. Authors can upload supporting documentation to indicate the use of appropriate reporting guidelines for health-related research (see EQUATOR Network), life science research (see the BioSharing Information Resource), or the ARRIVE guidelines for reporting work involving animal research. Where applicable, authors should refer to any relevant reporting standards documents in this form.

If you have any questions, please consult our Journal Policies and/or contact us: editorial@elifesciences.org.

Sample-size estimation

- You should state whether an appropriate sample size was computed when the study was being designed
- You should state the statistical method of sample size computation and any required assumptions
- If no explicit power analysis was used, you should describe how you decided what sample (replicate) size (number) to use

Please outline where this information can be found within the submission (e.g., sections or figure legends), or explain why this information doesn't apply to your submission:

Samples sizes were determined based on prior experience addressing these experimental designs and readout measures. Our standard operating procedure is that if an initial experience based estimation of sample size requirements results in a p value of 0.1 or below, exhibiting a trend toward significance, then the experiment is repeated with twice the initially estimated sample size. We did not encounter such instances in the submitted data sets. We also note that for multiple comparisons the software we use for analysis does not report specific p values but only >/< values and these are being reported. Sample sizes and the unit of measurement (e.g., axon, mitochondrion, etc) are reported in the Figure panels (within bars or otherwise denoted) and the figure legends, respectively. Information on statistical analysis is provided in the section titled "Quantification and statistical analysis".

Replicates

- You should report how often each experiment was performed
- You should include a definition of biological versus technical replication
- The data obtained should be provided and sufficient information should be provided to indicate the number of independent biological and/or technical replicates
- If you encountered any outliers, you should describe how these were handled
- Criteria for exclusion/inclusion of data should be clearly stated
- High-throughput sequence data should be uploaded before submission, with a private link for reviewers provided (these are available from both GEO and ArrayExpress)

Please outline where this information can be found within the submission (e.g., sections or figure legends), or explain why this information doesn't apply to your submission:

The “Quantification and statistical analysis” section describes biological replicates and provides the definition thereof for in vitro studies. The same section also describes the biological replicates for the in ovo/in vivo studies.

We did not encounter outlier in our data sets.

Statistical reporting

- Statistical analysis methods should be described and justified
- Raw data should be presented in figures whenever informative to do so (typically when N per group is less than 10)
- For each experiment, you should identify the statistical tests used, exact values of N, definitions of center, methods of multiple test correction, and dispersion and precision measures (e.g., mean, median, SD, SEM, confidence intervals; and, for the major substantive results, a measure of effect size (e.g., Pearson's r, Cohen's d)
- Report exact p-values wherever possible alongside the summary statistics and 95% confidence intervals. These should be reported for all key questions and not only when the p-value is less than 0.05.

Please outline where this information can be found within the submission (e.g., sections or figure legends), or explain why this information doesn't apply to your submission:

The "Quantification and statistical analysis" section details that all data sets are first analyzed for normalcy and if any data set in a comparison group fails the test then non-parametric analytical methods are used. Raw data are presented in figure panels when the presentation thereof is visually feasible without data points causing large clusters, and especially when not normally distributed. The statistical test applied for each comparison is denoted in the figure legend. For multiple comparisons the software we use for analysis does not report specific p values but only >/< values and these are being reported.

(For large datasets, or papers with a very large number of statistical tests, you may upload a single table file with tests, Ns, etc., with reference to sections in the manuscript.)

Group allocation

- Indicate how samples were allocated into experimental groups (in the case of clinical studies, please specify allocation to treatment method); if randomization was used, please also state if restricted randomization was applied
- Indicate if masking was used during group allocation, data collection and/or data analysis

Please outline where this information can be found within the submission (e.g., sections or figure legends), or explain why this information doesn't apply to your submission:

The "quantification and statistical analysis" section describes blinded analysis of data sets. Allocation of samples to experimental or control groups was random (e.g., for a set of 12 cultures, 6 would be allocated to control and 6 to experimental groups).

Additional data files ("source data")

- We encourage you to upload relevant additional data files, such as numerical data that are represented as a graph in a figure, or as a summary table
- Where provided, these should be in the most useful format, and they can be uploaded as "Source data" files linked to a main figure or table
- Include model definition files including the full list of parameters used
- Include code used for data analysis (e.g., R, MatLab)
- Avoid stating that data files are "available upon request"

Please indicate the figures or tables for which source data files have been provided:

eLife Sciences Publications, Ltd is a limited liability non-profit non-stock corporation incorporated in the State of Delaware, USA, with company number 5030732, and is registered in the UK with company number FC030576 and branch number BR015634 at the address 1st Floor, 24 Hills Road, Cambridge CB2 1JP | August 2014
